# Modulation of renal inflammation and tubular injury by calcitriol in patients with early diabetic kidney disease: a randomized controlled trial

**DOI:** 10.1080/07853890.2025.2577271

**Published:** 2025-10-27

**Authors:** Pringgodigdo Nugroho, Aida Lydia, Pradana Soewondo, Suhardjono, Ina Susianti Timan, Kuntjoro Harimurti, Zulkhair Ali

**Affiliations:** ^a^ Division of Nephrology and Hypertension, Department of Internal Medicine, Faculty of Medicine, Universitas Indonesia—Dr. Cipto Mangunkusumo Hospital, Jakarta, Indonesia; ^b^ Doctoral Program in Medical Sciences, Faculty of Medicine, Universitas Indonesia; ^c^ Division of Metabolic Endocrine, Department of Internal Medicine, Faculty of Medicine, Universitas Indonesia—Dr. Cipto Mangunkusumo Hospital, Jakarta, Indonesia; ^d^ Human Genetic Research Center IMERI, Faculty of Medicine, University of Indonesia; ^e^ Department of Clinical Pathology, Faculty of Medicine and Health Sciences, Universitas Kristen Krida Wacana UKRIDA, Jakarta; ^f^ Division of Geriatrics, Department of Internal Medicine, Faculty of Medicine, Universitas Indonesia—Dr. Cipto Mangunkusumo Hospital, Jakarta, Indonesia; ^g^ Division of Nephrology and Hypertension, Department of Internal Medicine, Faculty of Medicine, Universitas Sriwijaya, Indonesia

**Keywords:** Calcitriol, diabetic kidney disease, podocytopathy, tubulopathy, albuminuria, inflammation

## Abstract

**Introduction:**

Diabetic kidney disease (DKD) is marked by inflammation, fibrosis and oxidative stress. Calcitriol may offer renoprotective effects by modulating these processes. This double-blind, randomized controlled trial investigated the effects of calcitriol supplementation on kidney injury biomarkers in patients with early DKD.

**Patients and methods:**

This study randomized 120 patients with controlled type 2 diabetes, albuminuria, and estimated glomerular filtration rate above 45 mL/min/1.73 m^2^ to receive either 0.25 mcg/d calcitriol or a placebo for six months. The primary outcomes were changes in urinary nephrin, podocin, kidney injury molecule-1 (KIM-1), interleukin-6 (IL-6), and urinary albumin-to-creatinine ratio (UACR).

**Results:**

After 6 months, the calcitriol group showed lower increases in IL-6 levels (baseline 0.72 pg/mL to 0.87 pg/mL) compared to placebo (1.03 pg/mL to 2.94 pg/mL, *p* = 0.006), and lower KIM-1 levels (0.36 to 0.51 ng/mL vs. 0.55 to 0.99 ng/mL, *p* = 0.020). Full baseline and post-treatment values are provided in Tables 2. Urinary nephrin levels decreased in the calcitriol group, while change in the placebo group varied. Although UACR decreased more in the calcitriol group, the difference was not statistically significant (*p* = 0.099). Positive correlations were observed between KIM-1 and IL-6 in both groups, and a moderate correlation between IL-6 and urinary nephrin was observed in in the calcitriol group. No significant adverse events or changes in serum calcium or phosphate levels were reported.

**Conclusion:**

These findings suggest that Calcitriol supplementation may provide renoprotection in patients with early DKD by reducing inflammation and tubular injury, with potential effects on podocyte injury. Further research is required to validate these effects and determine the optimal dosing regimens.

**Trial registration:**

ClinicalTrials.gov (NCT05298163).

## Key messages

1. The administration of 0.25 mcg/d calcitriol for 6 months was safe and well tolerated, supporting its potential clinical applicability.
2. The protective effects of calcitriol in patients with early-stage DKD was primarily mediated through its anti-inflammatory properties, as evidenced by the significant reduction in urinary IL-6 and KIM-1 levels. The strong correlation between changes in IL-6 and KIM-1 further supports the anti-inflammatory property of calcitriol as a key mechanism underlying its renoprotective action.
3. However, the effects of calcitriol on podocyte injury and albuminuria are limited. This suggests that the benefits of 0.25 mcg/d calcitriol for 6 months benefits in patients with early DKD do not extend to direct podocyte protection.

## Introduction

Diabetes mellitus (DM) is one of the most significant global health challenges of the twenty first century. According to recent World Health Organization data, approximately 537 million adults are living with diabetes worldwide, with projections suggesting that this number will reach 643 million by 2030 [1]. Beyond hyperglycemia, DM leads to multiple complications including cardiovascular disease, neuropathy, and retinopathy, with diabetic kidney disease (DKD) being one of the most severe. In 2019, DKD contributed to over 130 million prevalent cases of chronic kidney disease (CKD) worldwide and accounted for more than 400,000 deaths, underscoring its major global impact. Strikingly, DM is the leading cause of both new and prevalent cases of CKD [2]. Individuals who develop DKD have significantly worse outcomes than those who do not [3]. Patients with DM face a dramatically increased risk of developing end-stage kidney disease (ESKD), with diabetes contributing to 30–50% of all new cases of kidney failure in developed countries [4,5].

The pathogenesis of DKD involves complex interactions between metabolic and hemodynamic pathways, leading to progressive kidney damage. Hyperglycemia triggers oxidative stress, advanced glycation end-products, and mitochondrial dysfunction, while glomerular hyperfiltration and activation of the renin–angiotensin–aldosterone system (RAAS) accelerates endothelial and podocyte injury [6]. Early detection and monitoring of DKD traditionally rely on clinical parameters and biochemical markers; however, there remains a critical need for more sensitive and specific biomarkers to identify kidney damage in its earliest stages and reliably track disease progression [7]. Notably, several podocyte-specific proteins, particularly nephrin, a key component of the glomerular slit diaphragm, and podocin, which are essential for proper podocyte function, have emerged as promising markers for glomerular injury and play crucial roles in maintaining glomerular filtration barrier integrity [8,9].

Inflammation is another critical pathway in the progression of DKD. Pro-inflammatory cytokines, especially interleukin-6 (IL-6), have demonstrated strong correlations with disease severity and progression [10]. T cells in individuals with type 1 DM exhibit heightened sensitivity to IL-6 due to increased IL-6 receptor expression [11]. In the context of DKD, IL-6 serves as a promising early marker of kidney damage, reflecting systemic inflammatory activity and early renal dysfunction rather than direct podocyte injury [12]. In contrast, podocyte injury is more specifically captured by podocyte-associated proteins such as nephrin and podocin. In addition, kidney injury molecule-1 (KIM-1), a transmembrane glycoprotein expressed in injured proximal tubular cells, is a specific indicator of tubular damage in patients with diabetes [13].

Current therapeutic approaches primarily focus on RAAS blockade using angiotensin-converting enzyme inhibitors (ACEi) or angiotensin receptor blockers (ARB) [14,15]. In the last decade, the discovery of sodium-glucose cotransporter 2 (SGLT2) inhibitors and non-steroidal mineralocorticoid receptor antagonists (nsMRAs) [16] has revolutionized DKD management [17]. These interventions form the cornerstone of treatment, effectively reducing blood pressure and albuminuria. However, many patients still experience disease progression and are at significant risk for cardiorenal disease [18–20]. This limitation has sparked interest in identifying complementary therapeutic strategies to enhance renoprotection in patients with DKD.

Vitamin D and its analogs have shown promising non-calcaemic effects in various kidney disease treatments [21,22]. Calcitriol (1,25-dihydroxyvitamin D3) has demonstrated potential in an experimental model of kidney disease [22,23]. Unlike synthetic analogs such as paricalcitol or doxercalciferol, calcitriol provides the most physiologically relevant activation of the VDR and has the advantage of being widely available, cost-effective, and clinically familiar in many healthcare settings. However, evidence regarding its renoprotective effects in patients with early-stage DKD remains limited, particularly concerning its effects on podocyte damage, tubular injury, and inflammatory markers in human subjects.

Therefore, in this study, we aimed to investigate the effects of calcitriol administration on key markers of kidney damage in patients with early DKD, focusing on podocyte injury (nephrin and podocin), tubular damage (KIM-1), and inflammation (IL-6). We hypothesize that calcitriol reduces urinary level of nephrin, podocin, KIM-1, albumin-to-creatinine ratio and IL-6. Furthermore, we propose that changes in IL-6 correlate with changes in nephrin, podocin, KIM-1 and albumin-to-creatinine ratio, highlighting calcitriol’s potential therapeutic role in the early management of DKD.

## Material and methods

### Study design and population

This double-blind, randomized controlled trial (RCT) was conducted at the Cipto Mangunkusumo and Pelni Hospitals in Jakarta, Indonesia, from July 22^nd^, 2022 to August 11^th^ 2023. This is a prospective study. The study protocol entitled ‘Efek Kalsitriol terhadap Podosit pada Pasien Penyakit Ginjal Diabetik: Kajian terhadap Podosin Urin, Nefrin Urin, Interleukin 6 Urin, KIM-1 Urin, Renin Plasma, dan Albuminuria’ [The Effect of Calcitriol in Diabetic Kidney Disease: A Study of Urinary Nephrin, Podocin, Albumin Creatinine Ratio, Interleukin-6, KIM-1, and Plasma Renin] was approved by the Ethics Committee of the Faculty of Medicine, Universitas Indonesia – Cipto Mangunkusumo National General Hospital, Jakarta, Indonesia (Ref: 176/UN2/F1/ETIK/PPM.00.02/2022; approval date: 21 February 2022). The trial was registered at ClinicalTrials.gov (NCT05298163). The study adhered to the principles of the Declaration of Helsinki, followed Good Clinical Practice guidelines and complied with the CONSORT guidelines for reporting clinical trials. All participants received verbal and written information about study objectives, procedures, potential risks, and benefits. A standardized, IRB-approved consent form was used. Trained research physicians obtained consent before enrollment.

Funding limitations delayed aspects of the study, contributing to an extension of the study period from the originally planned April–December 2022 to July 2022–August 2023 to ensure sufficient resources and follow-up time. Additionally, since early-stage CKD patients are rarely referred to Cipto Mangunkusumo Hospital (a tertiary care center), recruitment was slower than expected. To overcome this, Pelni hospital was added to expand the patient pool and include a broader range of CKD stages. These measures were necessary to meet the objectives of the study.

### Patient selection

To be eligible, participants must be adults aged 18–70 years with controlled type 2 diabetes (Hemoglobin [Hb] A1c < 8%), albuminuria (urinary albumin-to-creatinine ratio [UACR] > 30 mg/g), and an estimated glomerular filtration rate [eGFR] > 45 mL/min/1.73 m^2^. Exclusion criteria included uncontrolled hypertension despite ACEi/ARB treatment, hypercalcemia (total serum Calcium [Ca] > 10.5 mg/dL), hyperphosphatemia (serum phosphate > 5 mg/dL), hypersensitivity to calcitriol, and conditions causing proteinuria (urinary tract infection, urolithiasis, renal tuberculosis). Other exclusions included acute diabetes complications, recent acute coronary syndrome or stroke (within 6 months), chronic liver disease, malignancy, human immunodeficiency virus (HIV), systemic infections, malabsorption, alcoholism, smoking history, use of medications affecting calcitriol metabolism (thiazides, digoxin, anticonvulsants), pregnancy, and severe acute respiratory syndrome-Coronavirus-2 infection.

### Intervention protocol

Participants were randomly assigned to two equal groups (60 each) using SPSS^®^ version 25.0 (IBM Corp., Armonk, NY, USA). The intervention group received 0.25 mcg/d of calcitriol, while the control group received an identical placebo containing amylum, with no alternative supplement prescribed. To maintain blinding, both calcitriol and the placebo were encapsulated identically and dispensed monthly in unlabeled bottles.

Blinding was ensured at multiple levels:

- Participants: Group assignments were coded by an independent physician using SPSS, and the treatments were indistinguishable in appearance.
- Care Providers: Healthcare providers administering the intervention were unaware of treatment allocation, preventing bias in patient management.
- Researchers: The study remained blinded until analysis, with a separate research assistant responsible for coding the interventions, ensuring that investigators had no prior knowledge of group assignments.

The randomization code was secured and sealed by the independent physician until the study was completed, preserving the integrity of the trial.

The participants continued their standard DKD treatment, including the administration of ACEi/ARB or SGLT2 inhibitors. We visited monthly to monitor the blood pressure, glucose levels, and medication adherence. Participants were instructed to maintain their usual dietary and physical activity patterns for the duration of the study.

### Laboratory measurements

Clinical evaluations and laboratory assessments were performed at baseline, mid-study (month-3), and at the end of the study (month-6). Blood samples were collected after an overnight fast to measure HbA1c, calcium, phosphate, and creatinine levels. Urine samples were obtained using the midstream morning void technique and analyzed for podocin, nephrin, IL-6, KIM-1, and UACR.

Urinary podocin was quantified using the Human Podocin/PDCN (NPHS2) ELISA Kit (Abexxa Ltd., Milton, Cambridge, UK), which has a sensitivity (lower limit of detection) of 0.19 ng/mL and a detection range of 0.312–20 ng/mL. Podocin values were normalized to urinary creatinine to correct for urine dilution. Nephrin was measured using an Exocell ELISA kit (Exocell Inc., Philadelphia, Pennsylvania, USA), while IL-6, and KIM-1 using Quantikine^®^ ELISA kits (R&D Systems, Minneapolis, MN, USA). UACR was determined using Nycocard U-albumin (Alere Technologies AS, Norway) in a sandwich immunometric assay. All assays were performed according to the manufacturer’s protocol, with samples analyzed in duplicate. Intra- and inter-assay coefficients of variation were maintained below 10%.

Safety was monitored through monthly calcium and phosphate measurements, with predefined thresholds of serum calcium > 10.5 mg/dL and phosphate > 5.0 mg/dL. All adverse events were documented and evaluated for potential associations with study medication.

### Study endpoints

The primary endpoints were changes in urinary podocin, nephrin, KIM-1, and IL-6 levels and UACR from baseline to 6 months. The secondary endpoints were correlations between biomarkers. The safety endpoints included the incidence of hypercalcemia (serum Ca >10.5 mg/dL), hyperphosphatemia (>5.0 mg/dL), and other adverse events such as gastrointestinal symptoms, fatigue and allergic reactions. Hypercalcemia and hyperphosphatemia were assessed through monthly serum calcium and phosphate measurements using standard automated laboratory techniques. Clinical adverse events were documented through monthly anamnesis and physical examination.

### Sample size calculation

The sample size for this RCT was determined based on previously reported changes in urinary biomarkers in DKD intervention studies [24–29]. We calculated the required sample size using a significance level of α = 0.05 (corresponding to a critical value of 1.96) and 80% power (corresponding to a value of 0.842).

For each biomarker, we established clinically significant effect sizes based on existing literature. The expected effect sizes were: urinary podocin 7.5 ng/mmol (standard deviation 6.1 ng/mmol), urinary nephrin 538.7 ng/mmol (standard deviation 681 ng/mmol), urinary IL-6 50 pg/mmol (standard deviation 27 pg/mmol), UACR 18.56 mg/g (standard deviation 14.5 mg/g), and KIM-1 59.9 ng/mmol (standard deviation 37.6 ng/mmol). Among the candidate biomarkers, urinary nephrin required the largest sample size and was therefore selected as the primary determinant for sample size calculation. After accounting for a 10% dropout rate, 60 participants were enrolled in each group to ensure adequate power across all endpoints.

### Statistical analysis

Data were analyzed using SPSS^®^ version 25.0 (IBM Corp., Armonk, NY, USA). The Shapiro-Wilk test assessed normality. Baseline characteristics were reported as mean (standard deviation) or median (range) for normally and non-normally distributed data, respectively. Categorical variables are presented as numbers and percentages.

Comparisons between groups were conducted using unpaired t-tests for normally-distributed data and Mann-Whitney U tests for non-normally distributed data. Categorical variables were analyzed with chi-square or Fisher’s exact tests, as applicable. Changes over time were analyzed using repeated-measures analysis of variance or the Friedman test, based on data distribution.

Correlations between the parameters were determined using Spearman’s correlation coefficient. A *p-*value of < 0.05 was considered statistically significant. All tests were two-tailed.

## Results

### Participant flow

**Figure 1.**
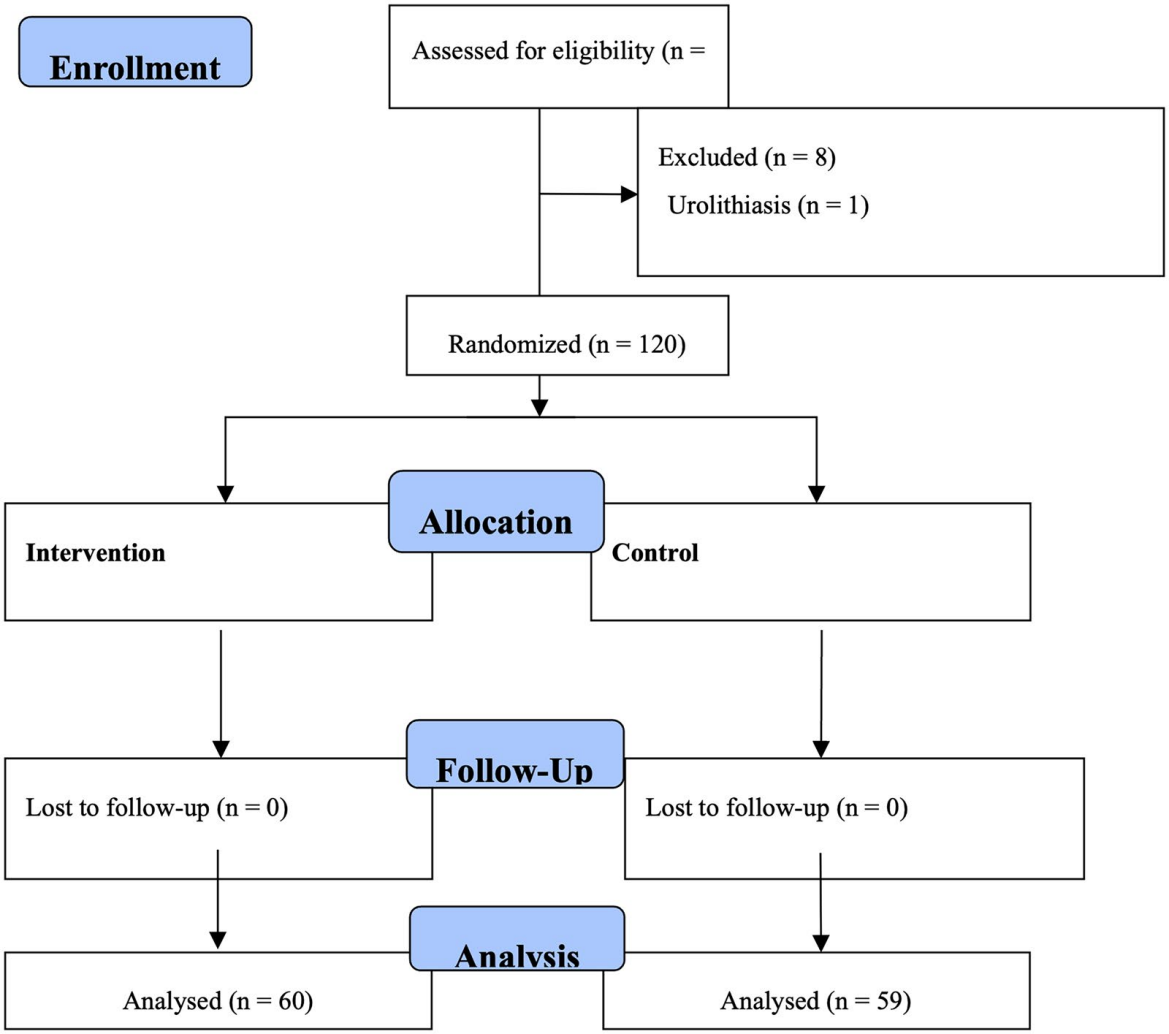
CONSORT chart.

Between July 22^nd^ 2022 and February 11^th^ 2023, we included 128 patients with DKD who met the inclusion criteria. We excluded eight patients: one was due to urolithiasis, five due to urinary tract infections, and two due to participation in other clinical studies. The remaining 120 patients were randomized into the calcitriol (*n* = 60) and placebo (*n* = 60) groups. One patient from the placebo group withdrew consent during this study period, resulting in the final analysis of 60 and 59 patients in the calcitriol and placebo groups, respectively (Figure 1).

### Recruitment

The study was conducted over a 6-month period. Monthly laboratory test for calcium and phosphate levels were performed to monitor therapy side effects. Other risk factors, such as hypertension and blood glucose, were managed through regular monthly monitoring, and adherence to routine medications. Laboratory tests for podocin, nephrin, UACR, eGFR, urinary IL-6, HbA1c, phosphate and urinary KIM-1 were performed at baseline (month 0), repeated mid-study (month 3) and at study completion.

### Baseline characteristics

All analyses were conducted based on the original assigned groups. The groups showed balanced demographic and clinical characteristics at baseline. The median age was 58.5 years in both groups (IQR: 51.25–63 years in the calcitriol group; 54–62.8 years in the placebo group). Female participants comprised 50% and 58.3% of the calcitriol and placebo groups, respectively. The median duration of diabetes was shorter in the calcitriol group (5 years, IQR: 2–10) than in the placebo group (8.5 years, IQR: 5–14).

Cardiovascular comorbidities were common in both groups. Hypertension was the most prevalent condition (81.7% and 76.6% in the calcitriol and placebo groups, respectively), followed by dyslipidemia (41.7% vs 46.7%). Coronary artery disease was present in 13.3% and 18.3% of patients in the calcitriol and placebo groups, respectively. Other comorbidities included cerebrovascular disease, peripheral arterial disease, and hyperuricemia, with similar distributions between both groups.

Medication use was comparable between the groups. ACEi/ARB therapy was prescribed to 58.3% and 65% of the patients in the calcitriol and placebo groups, respectively. Metformin was the most commonly used antidiabetic medication (70% and 65% in the calcitriol and placebo group, respectively), followed by sulfonylureas (56.7% vs 50%). The use of newer agents, such as SGLT2 inhibitors, was relatively low in both groups (6.7% vs. 1.7%) (Table 1).

**Table 1.** Baseline demographic and clinical characteristics.

	Calcitriol (*n* = 60) Mean (SD) / Median (IQR)	Placebo (*n* = 60) Mean (SD) / Median (IQR)	*P*
**Demographic**			
Age, years	58.5 (51.25–63)	58.5 (54–62.8)	0.891
Female, n (%)	30 (50)	35 (5.3)	0.464
Duration of DM, years	5 (2–10)	8.5 (5–14)	0.045
BMI, kg/m^2^	26.7 (5.1)	26.9 (5.1)	0.856
MAP, mmHg	98.5 (10.7)	99.2 (12)	0.308
**Comorbidities**			
Hypertension, n (%)	49 (81.7)	46 (76.7)	0.653
CHF, n (%)	4 (6.7)	2 (3.3)	0.675
CAD, n (%)	8 (13.3)	11 (18.3)	0.617
CVD, n (%)	4 (6.7)	1 (1.7)	0.361
PAD, n (%)	3 (5)	4 (6.7)	1.000
Dyslipidemia, n (%)	25 (41.7)	28 (46.7)	0.713
Hyperuricemia, n (%)	9 (15)	12 (20)	0.631
**Medications**			
ACEi / ARB, n (%)	35 (58.3)	39 (65)	0.573
CCB, n (%)	28 (46.7)	32 (53.3)	0.584
Beta blocker, n (%)	16 (26.7)	16 (26.7)	1.000
Diuretic, n (%)	3 (5)	3 (5)	1.000
Sulfonyluria, n (%)	34 (56.7)	30 (50)	0.583
Metformin, n (%)	42 (70)	39 (65)	0.697
TZD, n (%)	4 (6.7)	2 (3.3)	0.675
α-Glucosidase Inhibitor, n (%)	9 (15)	8 (13.3)	1.000
DPP4 Inhibitor, n (%)	27 (45)	30 (50)	0.493
SGLT2 Inhibitor, n (%)	4 (6.7)	1 (1.7)	0.361
Insulin, n (%)	25 (41.7)	25 (41.7)	1.000
Statin, n (%)	28 (46.7)	25 (41.7)	0.713
**Laboratory parameter**			
Nephrin, ng/mL	1150 (890–1874)	1538 (401–2574)	0.576
Podocin, ng/mL	.50 (.45–.63)	.52 (.45–0,62)	0.869
UACR, mg/g	116.1 (60–233.1)	150 (60–341.75)	0.324
KIM-1, ng/mL	.36 (.12–1.02)	.55 (.11–2.44)	0.358
IL-6, pg/mL	.72 (.31–2.89)	1.03 (.35–3.35)	0.529
Serum calcium, mmol/L	2.35 (0.1)	2.35 (0.12)	0.774
Serum phosphorus, mmol/L	1.29 (0.19)	1.26 (0.23)	0.751
Urea, mmol/L	5.6 (4.16–7.03)	5.4 (4.5–6.3)	0.755
Serum creatinine, μmol/L	88.4 (70.7–106)	88.4 (70.7–106)	0.939
eGFR, ml/min/1.73m^2^	70 (5725–8775)	66.5 (53.4–82.78)	0.270
Hb, g/dL	12.9 (1.5)	12.3 (1.7)	0.028
HbA1c, %	7.1 (6.2–7.6)	7 (6.42–7.48)	0.846
Serum uric acid, mmol/L	0.38 (0.08)	0.38 (0.09)	0.945
LDL, mmol/L	2.7 (2.2–3.6)	2.97 (2.4–3.5)	0.176
Triglyceride, mmol/L	1.5 (1.1–2.3)	1.7 (1.3–2.4)	0.171

BMI: body mass index, MAP: mean arterial pressure, CHF: chronic heart failure, CAD: coronary artery disease, CVD: cerebrovascular disease, PAD: peripheral arterial disease, ACEi: Angiotensin converting enzyme inhibitor, ARB: angiotensin receptor blocker; CCB: calcium channel blocker; TZD: thiazolidinediones; DPP4: dipeptidyl peptidase-4, SGLT2: sodium/glucose cotransporter-2; UACR: urinary albumin creatinine ratio, KIM-1: kidney injury molecule-1, IL-6: interleukin-6; eGFR: estimated glomerular filtration rate; Hb: hemoglobin; HbA1c: hemoglobin A1c, LDL: low-density lipoprotein.

### Laboratory parameters at baseline

The baseline laboratory parameters were generally similar between the groups, with a few notable differences. The median UACR was lower in the calcitriol group (116.1 mg/g, IQR: 60–233.1) than in the placebo group (150 mg/g, IQR: 60–341.75). The median urinary nephrin levels were also lower in the calcitriol group (1150 ng/mL, IQR: 890–1874) than in the placebo group (1538 ng/mL, IQR: 401–2574); however, this difference was not statistically significant.

Other key baseline parameters included similar median HbA1c levels (7.1% vs 7.0%), eGFR (70 vs 66.5 mL/min/1.73m^2^), and serum calcium (9.4 mg/dL in both groups) (Table 1).

### Changes in biomarkers over the study period

After 6 months of treatment, several patterns emerged in the biomarker trajectories. Baseline and post-treatment numerical values for all biomarkers (nephrin, podocin, UACR, KIM-1, and IL-6) are detailed in Tables 2, in addition to the reported changes. Table 3 shows the direct comparison of biomarker values between groups at the end of the 6-month intervention.

**Table 2.** Laboratory value at month 0, 3 and 6.

	Calcitriol (*n* = 60) Mean (SD) / Median (IQR)					Placebo (*n* = 60) Mean (SD) / Median (IQR)					
	T0	T3	T6	Δ_TC_	95% CI	T0	T3	T6	Δ_TP_	95% CI	*p* *
**Nephrin**, ng/mL	1150 (890–1874)	1022 (346–2035)	884 (220–2087)	−326 (−1152–1063)	(−828–.651)	1538 (401–2574)	717 (181–1538)	1170 (703–3148)	−152 (−1505–1054)	(−690–350)	.871
**Podocin** ng/mL	.5 (.45–.63)	.64 (.56–.76)	.54 (.49– .65)	.03 (−0.07–.19)	(.01–.09)	.52 (.45-.62)	.63 (.5–.8)	.52 (.48–.58)	.01 (−0.08–.11)	(−0.04–.59)	.404
**UACR**, mg/g	116.05 (60–233.1)	51.2 (16.82–144.31)	39.25 (19.16–161.09)	−37.63 (−138.98– 13.87)	−61.26–-15.77	150 (60–341.75)	72.45 (13.45–988.68)	118.3 (14.5–1264.53)	−22.17 (−74.77–380.49)	−49.7–42.51	.099
**KIM-1**, ng/mL	.36 (.12–1.02)	.65 (.29–1.81)	.51 (.2–1.56)	.02 (−0.38–.8)	(−0.06–.26)	.55 (.11–2.44)	.86 (.19–2.52)	.99 (.31–2.86)	.28 (−0.15–1.01)	(−0.03–.66)	.199
**IL-6,** pg/mL	**.72** **(.31–2.89)**	.59 (.28–5.47)	**.87** **(.39–4.58)**	**.12** **(−0.72–1.33)**	**(−0.7–.43)**	**1.03** **(.35–3.35)**	1.68 (.48–5.56)	**2.94** **(1.13–9.92)**	**1.60** **(−0.05–4.99)**	**(.28–2.53)**	**.006**
**HbA1c**, %	7.1 (6.2–7.6)	6.9 (6.1–7.5)	7 (6–7.8)	0 (−0.4–.4)	−0.1–.15	7 (6.4–7.5)	7.1 (6.3–7.8)	7.5 (6.8–8)	0.4 (0–1.1)	.1–.7	.003
**Serum calcium**, mmol/L	2.35 (0.1)	2.32 (2.2–2.4)	2.33 (0.1)	0 (−0.1–0.07)	(−0.3–.1)	2.35 (2.3–2.4)	2.3 (2.2–2.4)	2.3 (2.2–2.4)	−0.2 (−0.1–0.025)	(−0.35– −0.1)	.261
**Serum phosphorus**, mmol/L	1.3 (.2)	1.3 (1.1–1.4)	1.27 (.2)	−0.35 (.55)	(−0.18–.10)	1.3 (1.3–1.4)	1.2 (1.1–1.4)	1.2 (1.1–1.4)	−0.08 (.65)	(−0.25–.08)	.656

UACR, urinary albumin to creatinine ratio; KIM-1, kidney injury molecule-1; IL-6: interleukin-6.

Δ_TC_ = The change in biomarker levels in those receiving calcitriol, calculated as the difference between levels measured at month 6 and baseline (month 0).

Δ_TP_ = The change in biomarker levels in those receiving placebo, calculated as the difference between levels measured at month 6 and baseline (month 0).

* *p* = difference between Δ_TC_ and Δ_TP_ (Mann-Whitney U).

**Table 3.** Laboratory parameter comparison at sixth month.

	Calcitriol (*n* = 60)		Placebo (*n* = 60)		*p*
	Mean (SD) / Median (IQR)	95% CI	Mean (SD) / Median (IQR)	95% CI	
**Nephrin**, ng/mL	884 (220–2087)	535–1376	1170 (703–3148)	919–1620	.106
**Podocin**, ng/mL	.54 (.49–.65)	.51–.57	.52 (0.48–0.58)	.51–.55	.316
**UACR**, mg/g	39.25 (19.16–161.09)	25.4–88.1	118.3 (14.5–1264.53)	29.76–378	.186
**KIM-1**, ng/mL	**.51** **(.20–1.56)**	**.33–.75**	**.99** **(.31–2.86)**	**.81–2.1**	**.020**
**IL-6,** pg/mL	**.87** **(.39–4.58)**	**.63–1.93**	**2.94** **(1.13–9.92)**	**2.31–5.1**	**.012**

UACR: urinary albumin to creatinine ratio; KIM-1: kidney injury molecule-1; IL-6: interleukin-6.

1. *Urinary Nephrin and Podocin:* The calcitriol group showed a consistent decrease in urinary nephrin levels from baseline to 6 months (1150–884 ng/mL). In contrast, the placebo group showed more variable changes, ending at 1170 ng/mL. Podocin levels showed minimal variation between the groups at the end (0.54 vs 0.52 ng/mL).
2. *Albuminuria:* UACR decreased more in the calcitriol group (116.05–39.25 mg/g) compared to placebo (150–118.3 mg/g), although the difference was not statistically significant (95% CI −61.26 – −15.77, p = 0.099).
3. *Inflammatory and Injury Markers:* The most striking differences were observed in IL-6 and KIM-1 levels. IL-6 showed significantly lower increases in the calcitriol group (median change: 0.12 pg/mL) compared to the placebo group (median change: 1.60 pg/mL, p = 0.006). Similarly, the final KIM-1 levels were significantly lower in the calcitriol group (0.51 vs 0.99 ng/mL, p = 0.020).

### Correlation analysis

Spearman correlation analysis identified several significant associations between biomarkers following 6 months of treatment:

1. KIM-1 and IL-6 levels demonstrated a strong positive correlation in both the calcitriol (r = 0.677, p < 0.001) and placebo groups (r = 0.612, p < 0.001), suggesting a consistent relationship between tubular injury and inflammation across treatments.
2. In the calcitriol group, IL-6 levels showed a moderate positive correlation with urinary nephrin levels (r = 0.342, p = 0.007), a relationship not observed in the placebo group (r = 0.090, p = 0.497).

### Safety and tolerability

Monthly monitoring of safety parameters showed excellent tolerability of calcitriol supplementation. No significant changes were observed in serum calcium (baseline: 9.4 ± 0.4 mg/dL; 6 months: 9.36 ± 0.55 mg/dL) or phosphate levels (baseline: 3.98 ± 0.59 mg/dL; 6 months: 3.94 ± 0.56 mg/dL) in the calcitriol group. No participants developed hypercalcemia or hyperphosphatemia, requiring treatment discontinuation (Table 2). No cases of hypercalcemia, hyperphosphatemia, or treatment-related adverse events were observed over six months of calcitriol therapy. In addition, no participants reported gastrointestinal symptoms (nausea, vomiting, abdominal pain, constipation, or diarrhea), generalized symptoms (fatigue, excessive thirst, polyuria), cardiac disturbances, or hypersensitivity reactions (rash, pruritus, or dyspnea).

The completion rate was high, with one participant discontinuing the study, suggesting a good tolerability of the intervention. No significant adverse events attributed to the study medication were reported throughout the 6-month treatment period. A summary is provided in Supplementary Table 1.

## Discussion

We investigated the effects of 6-month calcitriol supplementation on multiple kidney injury biomarkers in patients with early DKD. These findings demonstrate significant reductions in inflammatory and tubular injury markers, with potential effects on podocyte injury markers.

### Effects of calcitriol on kidney biomarkers

#### Podocyte injury

##### Nephrin

Calcitriol (0.25 mcg/d) did not significantly reduce urinary nephrin levels compared to placebo (Table 4). However, the treatment group showed a steady decline in nephrin levels across the study period. Since nephrin loss is linked to worsening proteinuria and renal decline, even modest reductions may hold biological relevance.

**Table 4.** The changes in biomarker levels in the calcitriol and placebo groups.

	Calcitriol (*n* = 60)		Placebo (*n* = 59)		*p*
	Mean (SD) / Median (IQR)	95% CI	Mean (SD) / Median (IQR)	95% CI	
**A.Nephrin (ng/mL)**					
Baseline	1150 (890–1874)	1065–1550	1530 (401–2574)	972–1936	.215
3rd month	1022 (346–2035)	6641–1310	717 (181–1538)	532–1108	
6th month	884 (220–2087)	535–1376	1170 (703–3148)	919–1620	
**B.Podocin (ng/mL)**					
Baseline	.50 (.45–.63)	.46–.55	.52 (.45–.62)	.48–.56	.427
3rd month	.64 (.56–.76)	.60–.66	.63 (.5–.8)	.58–.7	
6th month	.54 (.49–.65)	.51–.57	.52 (.48–.58)	.51–.55	
**C.UACR (mg/g)**					
Baseline	118.4 (60–233.1)	80.0–167.0	150 (60–341.75)	90.25–170	.225
3rd month	57.5 (16.82–144.31)	39.5–96.3	72.45 (13.45–988.68)	20.9–227.3	
6th month	39.25 (19.16–161.09)	25.4–88.1	118.3 (14.5–1264.53)	29.76–378	
**D.KIM-1 (ng/mL)**					
Baseline	.36 (.12–1.02)	.23–.71	.53 (.11–2.44)	.25–1.4	.146
3rd month	.65 (.29–1.81)	.43–.89	.86 (.19–2.52)	.53–1.5	
6th month	.51 (.2–1.56)	**.33–.75**	.99 (.31–2.86)	**.81–2.1**	
**E.IL-6 (pg/mL)**					
Baseline	.72 (.31–2.89)	.41–1.3	1.03 (.35–3.35)	.5–1.9	.030
3rd month	.59 (.28–5.47)	.40–1.4	1.68 (.48–5.56)	.95–2.45	
6th month	.87 (.39–4.58)	**.63–1.93**	2.94 (1.13–9.92)	**2.31–5.1**	

UACR: urinary albumin to creatinine ratio; KIM-1: kidney injury molecule-1; IL-6: interleukin-6.

Nephrin expression is known to decrease under hyperglycemic conditions compared to normal glucose levels (*p* < 0.05) [30]. Experimental studies have shown that vitamin D can inhibit fibronectin production in mesangial cell cultures, increases nephrin expression in podocyte cultures [23]. Animal studies have shown that nephrin and podocin expression were enhanced in diabetic animals treated with 22-oxa-calcitriol [31]. Our trial is among the first to evaluate the effect of calcitriol on nephrin in human DKD.

These lack of significance may reflect limited study power, a relatively low dose or the short 6-month duration. Higher doses, multiple doses, or longer observation periods may be required to achieve statistically significant differences in nephrin levels. Podocyte injury in DKD is complex and nephrin expression can vary depending on disease stage and conditions [32].

Importantly, stabilization or even small, non-significant improvements in nephrin may still have implications for long-term renal protection, since podocyte loss is a critical determinant of irreversible progression to ESKD. Larger and longer trials are needed to determine whether calcitriol can slow podocyte depletion and thereby modify long-term outcomes.

##### Podocin

Calcitriol did not significantly lower urinary podocin levels compared to the placebo (Table 4). Similar findings were reported by Timm et al. who found no significant changes in podocyte and nephrin mRNA expression following vitamin D supplementation in patients with CKD [33]. Animal studies also suggested potential protective effects of calcitriol on podocyte integrity [34]. The lack of change in our study may reflect the relative stability of podocyte injury in early DKD, as podocin alterations are more prominent in advanced disease. In patients with milder kidney damage, calcitriol may not substantially affect podocin levels. Its renoprotective effects could be more evident when podocyte injury is strongly linked to increased podocin excretion. Furthermore, the low urinary concentration and small molecular size of podocin (42 kDa) [35] may limit its reliability as a clinical biomarker.

Nevertheless, stabilization of podocin levels without further increase may suggest a protective trend. From a long-term perspective, preventing progressive rises in podocin excretion could help preserve podocyte integrity, which is key to delaying proteinuria and slowing renal decline. Future research should investigate this relationship in larger populations, particularly in patients with advanced DKD and higher albuminuria, to clarify the potential renoprotective role of calcitriol.

##### Tubular injury and KIM-1

Calcitriol treatment significantly lowered KIM-1 levels after 6 months compared to placebo (Table 4). KIM-1 is a key biomarker of DKD progression. It mediates the uptake of palmitic acid-bound albumin, leading to DNA damage, interstitial inflammation, and tubular fibrosis [36]. Abid-Khan et al. reported that KIM-1 increases with progressive kidney injury, even before renal function declines [37]. The reduction observed in our study a tubular protective effect of calcitriol, which may have long-term benefits, even though our study duration was insufficient to capture hard clinical outcomes. These findings are consistent with experimental studies showing that calcitriol reduces KIM-1 expression and mitigates tubular fibrosis [38]. To our knowledge, this is the first trial to evaluate the impact of calcitriol on KIM-1 expression in DKD patients, offering new clinical evidence for its renoprotective properties.

##### Inflammatory response and IL-6

Inflammatory cytokine levels, including IL-6, were significantly elevated in patients with poorly controlled DM [39]. It is particularly important because IL-6 is a key mediator of inflammation in patients with DKD [40].

Calcitriol significantly reduced IL-6 levels in our trial, suggesting that anti-inflammatory effects may be central to its protective action.This finding aligns with experimental evidence suggesting that vitamin D analogs modulate inflammatory pathways [41]. Although our trial was not designed to assess clinical endpoints, the observed suppression of IL-6 provides mechanistic support for calcitriol as a potential adjunctive therapy to slow DKD progression.

##### Urine albumin-creatinine ratio

Calcitriol led to a non-significant reduction in UACR, with only numerical improvements observed. The modest dose and 6-month duration may have been insufficient to produce a measurable reduction in proteinuria. These findings align with prior studies reporting similar non-significant changes with calcitriol, either as a monotherapy or as an adjunct to other treatments [42,43]. A meta-analysis, however, showed that higher vitamin D doses can reduce proteinuria and may exert renoprotective effects by modulating albuminuria progression [44]. This highlights the need for higher doses, multiple-dosing regimens, and longer follow-up periods to confirm beneficial effects on albuminuria.

#### Correlation analysis and mechanistic insights

We observed a strong correlation between IL-6 and KIM-1 levels in both groups, highlighting the link between inflammation and tubular injury. Interestingly, IL-6 and nephrin correlated only in the calcitriol group, suggesting that it may indirectly influence podocyte injury *via* inflammatory pathways (Table 5).

**Table 5.** The correlation between change in urinary IL-6 with urinary podocin, nephrin, ACR, and KIM-1 in both treatment groups.

		IL-6	
		Calcitriol (*n* = 60)	Placebo (*n* = 59)
Podocin	r	.055	.058
	P	.677	.665
Nephrin	r	**.342**	.090
	P	**.007**	.497
UACR	r	−0.301	< .001
	P	.190	.999
KIM-1	r	**.677**	**.612**
	P	**< .001**	**< .001**

UACR: urinary albumin to creatinine ratio; KIM-1: kidney injury molecule-1; IL-6: interleukin-6.

Our results suggest that calcitriol primarily protects against tubular injury rather than podocyte injury. This is supported by the significant reduction in KIM-1 levels and suppression of IL-6 in the calcitriol group compared to placebo. The strong IL-6–KIM-1 association highlights that calcitriol’s anti-inflammatory properties are critical in preventing tubular damage. Our findings extend previous research by providing clinical evidence for the effects of calcitriol on multiple kidney injury pathways [45].

Elevated urinary KIM-1 is known to increase kidney damage and worse outcomes through proximal tubular signaling and macrophage activation. This leads to the expression of cytokines and chemokines that attract neutrophils to the injury site, thereby amplifying the local inflammatory response, hypoxia, and cell damage [46,47].

Previous studies focused primarily on proteinuria or single markers. However, our comprehensive biomarker analysis provides deeper insights into the potential mechanisms of action. The significant effects on IL-6 and KIM-1 align with experimental studies [48] and provide novel evidence in patients with DKD.

In contrast, no significant effects were observed on podocin and nephrin, indicating limited influence on podocyte injury. Although nephrin and podocin did not change significantly, their stabilization may still hold biological relevance, since progressive podocyte loss is strongly linked to long-term renal function decline. This presents a grey area that warrants further investigation. These findings are particularly relevant, given the growing recognition of podocyte injury as a key mechanism in DKD progression [49,50]. It is possible that calcitriol acts first on inflammatory and tubular pathways, with downstream effects on podocytes potentially requiring longer treatment duration, higher doses, or larger study populations to be detected.

**Figure 2.**
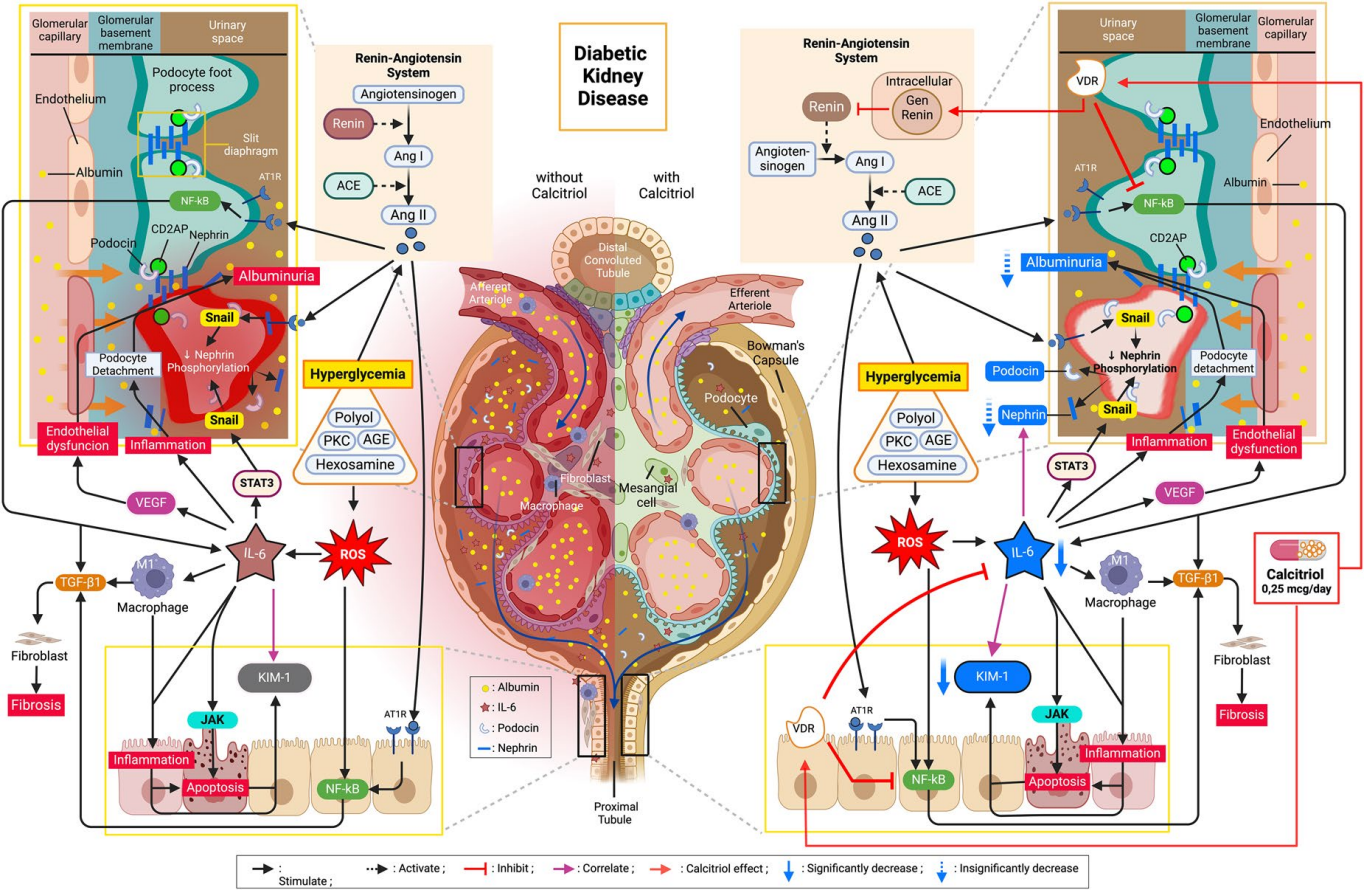
Proposed Mechanism of the Renoprotective Properties of Calcitriol in DKD (Created with BioRender.com). Chronic hyperglycemia activates multiple metabolic pathways (polyol, PKC, AGE, and hexosamine) leading to oxidative stress (ROS) and podocyte as well as tubular injury. In podocytes, oxidative stress and RAAS activation promote NF-κB signaling, nephrin phosphorylation, and CD2AP disruption, resulting in detachment of foot processes, albuminuria, and progressive endothelial dysfunction. Snail-mediated pathways contribute further to podocyte damage. In the proximal tubules, hyperglycemia-induced ROS, IL-6/STAT3 signaling, and macrophage activation increase the expression of KIM-1, promoting apoptosis, inflammation, and fibrosis via TGF-β1 and VEGF. Calcitriol, through vitamin D receptor (VDR) activation, exerts multiple protective effects. It suppresses NF-κB and STAT3 signaling, reduces IL-6 production, and lowers KIM-1 expression, thereby attenuating tubular injury, inflammation, and apoptosis. Calcitriol also helps preserve podocyte integrity by stabilizing nephrin and podocin expression, reducing foot process detachment, and limiting albuminuria. Overall, calcitriol acts on both inflammatory and structural pathways, targeting podocyte injury, tubular damage, and RAAS-mediated effects, suggesting its role as a potential adjunctive therapy in early DKD. Abbreviations: AGE: advanced glycation end-products; PKC: protein kinase C; ROS: reactive oxygen species; NF-κB: nuclear factor-κB; RAAS: renin–angiotensin–aldosterone system; IL-6: interleukin-6; STAT3: signal transducer and activator of transcription-3; KIM-1: kidney injury molecule-1; TGF-β1: transforming growth factor-β1; VEGF: vascular endothelial growth factor; VDR: vitamin D receptor.

Based on our findings, we propose that calcitriol exerts its renoprotective effects in patients with DKD through multiple pathways: 1) direct anti-inflammatory effects through IL-6 inhibition, 2) protection against tubular injury, as evidenced by reduced KIM-1 levels, and 3) potential podocyte stabilization through the modulation of inflammatory pathways. The significant correlation between IL-6 and KIM-1 suggests that inflammatory pathways are central to the protective effects of calcitriol (Figure 2).

#### Safety and adverse event

No significant changes were observed during the monthly monitoring of calcium and phosphate levels throughout the study period, confirming the safety of 0.25 mcg/d calcitriol supplementation. This aligns with the findings of previous studies demonstrating the safety of low-dose calcitriol in patients with CKD [51,52]. The absence of hypercalcemia or hyperphosphatemia in our study population suggests that this dosing regimen is well-tolerated by patients with early DKD.

#### Calcitriol vs. other DKD therapy

This study highlights calcitriol’s potential renoprotective effects, particularly in reducing tubular injury markers. However, its effect should be interpreted in the context of current DKD therapies. SGLT2-inhibitors have shown robust benefits in reducing proteinuria and improve renal outcomes through hemodynamic, metabolic and anti-inflammatory effects [53–55]. Finerenone, an nsMRA, have also demonstrated robust reduction in inflammation, fibrosis and albuminuria [56–59].

While calcitriol may not match the efficacy of these agents, its ability to target inflammation and tubular injury could provide additional benefits before significant podocyte damage occurs. These findings suggest a potential of calcitriol as an adjunctive therapy in early DKD. Further studies are needed to clarify the optimal use of calcitriol in early disease stages, and its synergy with the established therapies.

#### Clinical implications and future directions

Our findings have important clinical implications. The reductions in IL-6 and KIM-1 suggest that calcitriol may help slow pathways closely linked to long-term DKD progression and adverse renal outcomes. Although our study did not assess eGFR decline or ESKD events, the biomarker changes provide hypothesis-generating evidence that calcitriol could support long-term kidney preservation.

It is important to note that all participants in our study received standard-of-care therapies, and calcitriol or placebo was administered in addition. Therefore, our findings should not be interpreted as a head-to-head comparison with established therapies. Instead, the results provide exploratory evidence that calcitriol may complement current treatment regimens by targeting inflammatory and tubular pathways [60,61]. This may be particularly relevant for patients with elevated levels of inflammatory markers or early tubular injury. While SGLT2 inhibitors and finerenone have demonstrated robust renoprotective effects, calcitriol may offer additional benefits through different mechanisms. Future trials should investigate whether calcitriol provides additive or synergistic effects when combined with these cornerstone therapies. The favorable safety profile of 0.25 mcg/day supports its potential integration into current treatment protocols; however, careful patient selection and monitoring remain essential.

Future research should focus on evaluating different calcitriol dosing regimens, identifying patient subgroups most likely to benefit from treatment, investigating longer-term outcomes, exploring combination therapies with other renoprotective agents, investigating biomarker-guided therapy using IL-6 or KIM-1 levels to identify patients most likely to benefit from calcitriol, evaluating combination therapies, particularly with SGLT2 inhibitors or glucagon-like peptide-1 receptor agonists, long-term studies to assess the impact on hard clinical endpoints, and developing targeted vitamin D analogs with enhanced renoprotective properties.

#### Study strengths and limitations

This double-blind, randomized controlled trial has several strengths. The study design minimized bias and enhanced the reliability of the results through proper randomization and blinding. The inclusion of multiple biomarkers provides comprehensive insights into the various pathways affected by calcitriol in patients with early DKD. The 6-month duration allowed adequate time to observe the treatment effects, and the high completion rate (99.2%) strengthened the validity of our findings. This trial also enrolls randomly selected participants, ensuring that the study population reflects real-world patients who would receive calcitriol in clinical practice, enhancing the applicability of this study.

This study has several limitations. First, the use of a single calcitriol dose (0.25 mcg/day) and a 6-month duration limited assessment of dose–response and long-term outcomes such as eGFR decline. Second, while the sample size was adequate for primary endpoints, larger studies are needed to validate secondary findings. Third, no formal multiplicity correction was applied; therefore, our biomarker results should be considered exploratory. Finally, dietary vitamin D intake and baseline 25(OH)D levels were not assessed; although calcitriol directly activates the VDR, variability in nutritional vitamin D status may still have influenced responses. Future larger and longer studies are warranted to address these gaps.

## Conclusion

Calcitriol 0.25 mcg/d for 6 months significantly reduced markers of tubular injury and inflammation, with potential protective effects on podocyte injury.. These findings suggest the role of calcitriol in mitigating early DKD progression.

## Supplementary Material

CONSORT 2010 Checklist Revised.doc

Supplementary Files_Table of Adverse Events.docx

## Data Availability

Data available on request from the authors. For any inquiries regarding the data used in this study, please contact the corresponding author Pringgodigdo Nugroho through email.
