# Supplementary material for: Modulation of renal inflammation and tubular injury by calcitriol in patients with early diabetic kidney disease: a randomized controlled trial
Source: Ann Med. 2025 Oct 27;57(1):2577271. doi: 10.1080/07853890.2025.2577271 (PMC12570221; doi:10.1080/07853890.2025.2577271)
Supplement: Supplementary Files_Table of Adverse Events.docx [file IANN_A_2577271_SM8152.docx]

**Supplementary Files**

**Supplementary Table 1. Summary of Adverse Events Over 6 Months**

| **Adverse Event Type** | **Calcitriol (n=60)** | **Placebo (n=60)** | **Total (N=120)** |
| --- | --- | --- | --- |
| Hypercalcemia (serum Ca >10.5 mg/dL) | 0 | 0 | 0 |
| Hyperphosphatemia (serum Phosphate >5.0 mg/dL) | 0 | 0 | 0 |
| Gastrointestinal symptoms* | 0 | 0 | 0 |
| Fatigue | 0 | 0 | 0 |
| Hypersensitivity reactions† | 0 | 0 | 0 |
| Other adverse events | 0 | 0 | 0 |
| **Any adverse event** | 0 | 0 | 0 |
| *Gastrointestinal symptoms included nausea, vomiting, abdominal pain, constipation, and diarrhea.  †Hypersensitivity reactions included rash, pruritus, or dyspnea.  Note: No participants discontinued due to adverse events. | | | |
